# Strategies for positive leadership attitude among operational managers post-COVID-19 at primary health care clinics in the City of Johannesburg

**DOI:** 10.4102/safp.v67i1.6157

**Published:** 2025-09-16

**Authors:** Neo Khakhau, Elizabeth M. Nkosi, Sanele E. Nene

**Affiliations:** 1 Department of Nursing, Faculty of Health Sciences, University of Johannesburg, Johannesburg, South Africa

**Keywords:** strategies, leadership, attitudes, COVID-19, primary health care clinics

## Abstract

**Background:**

The quality of the leadership shown by operational managers was tested during coronavirus disease 2019 (COVID-19). They were expected to demonstrate positive leadership attitudes while being stretched by the demands of the pandemic.

**Methods:**

A qualitative, descriptive, phenomenological approach was used in this study to explore and describe strategies that can enhance and sustain a positive leadership attitude among operational managers after the COVID-19 pandemic. Ten operational managers were purposively sampled from primary health care (PHC) clinics in different regions of the City of Johannesburg. Data were collected using semi-structured individual interviews. Colaizzi’s seven-step descriptive phenomenological analysis method was used to analyse and summarise the data to the point of data saturation.

**Results:**

Three themes emerged on ways to enhance and sustain positive leadership attitudes among operational managers in this study: (1) debriefing and counselling services, (2) teamwork, team-building and senior management support and (3) continuous staff development.

**Conclusion:**

The study suggests strategies to enhance and sustain positive leadership attitudes after COVID-19. This will prepare operational managers for future pandemics or crisis situations.

**Contribution:**

This is the first study in the City of Johannesburg that focuses on strategies to enhance and sustain positive leadership attitudes among operational managers at PHC clinics in the City of Johannesburg. The findings will provide leadership with direction on the requirements for support, equipment and transparency for subordinates should a pandemic or crisis occur in future.

## Introduction

Attitudes have been described as ‘psychological tendencies that are expressed by evaluating a particular entity with some degree of favor or disfavor’.^1^ Literature affirms that attitudes signify one’s positive or negative appraisal of a person, idea or object, articulated through feelings, beliefs and behaviours.^2^ Leadership qualities refer to the inherent traits and skills that distinguish a leader, while leadership attitude describes a leader’s mindset and approach to situations. Qualities are frequently perceived as more initial and personality-driven, while attitude is a more vibrant and conscious choice’. In essence, ‘an attitude comprises of how we feel, what we think, and what we are persuaded to do about something’.^1^

Good leaders should have positive attitudes about their work environment and should spread these attitudes to their followers, as this can create a conducive working environment.^3^ Ultimately, the nursing profession is about caring, and operational managers must demonstrate positive, caring attitudes as one of their leadership attributes.^4^ Good leadership qualities for modelling include self-awareness, credibility and humility. A good leader is constantly aware of their actions, displays humility, cultivates honesty and a trustworthy environment in an open, communicative space to ensure ideal staff and organisational performance.^3^

Leadership is the process or ability to enthusiastically influence behaviour, and to motivate and direct subordinates to attain the organisation’s objectives as defined by its strategic plan.^5^ Managers are expected to be leaders and should consequently demonstrate positive leadership attitudes and attributes to effectively drive organisational change.^6^ However, there is a difference between management and leadership roles, and often these two are confused. Leaders inspire, empower, mentor and motivate, while managers direct, plan, organise and control activities, whether at the operational or executive level.^7^

Positive leadership attitudes are important at all levels of care, particularly in a decentralised system like primary health care (PHC), where competency is required to successfully perform critical functions of care.^8^ Leadership attitudes are important to enhance essential competencies in PHC performance.^8^ When operational managers’ leadership attitudes are negative, the quality of care provided in PHC is affected, and users of these services become disgruntled and lose trust in the system.^7^

According to literature, some examples of negative leadership attitudes include the invasion of staff privacy and being reminded of their past errors and failures, which dents their egos and deters them from personal and professional development.^7^ Leadership is about resilience, taking decisive action, talking broadly about what should be carried out and being accountable for your actions. Leadership development is supported by the ability to adapt to changing circumstances and remain humble, focusing on building relationships and having good communication skills. Leadership development refers to an invariably positive gesture, which is imperative to enhance institutional competence and capability in leadership, to develop motivation and engagement, or to improve performance. Such positive attitudes are nurtured among PHC operational managers during development programmes to ensure that they remain truthful to the institutional values and culture. However, these attitudes were tested during the coronavirus disease 2019 (COVID-19) pandemic.

During the COVID-19 pandemic, PHC operational managers were under extraordinary pressure because of the critical roles they played in response to the demands of the pandemic. Their leadership attitudes were critical to ensuring the lives of the health users and to see to it that health care workers were safe and protected.^9^ They had to adjust their leadership attitudes to effectively manage the pandemic, which was abrupt, constantly evolving and psychologically and mentally consuming, so they could grow from being fragile to gaining resilience.^10^ The COVID-19 pandemic exposed multiple dysfunctions in the South African health system, such as shortages of staff, supplies and protective gear. This resulted in deaths among health care workers.^10^ To survive this difficult time, operational managers in PHC settings had to take a stance and demonstrate positive leadership attitudes to ignite hope and encourage nurses and supporting staff.^10^ The leadership worked tirelessly to guide staff cohesion and group work, and to maintain efficiency because of escalating patient numbers and staff deaths, increasing shortage of equipment, and fear of the unknown. This study explored and described the strategies that could enhance and sustain the positive leadership attitudes of operational managers’ leadership after the COVID-19 pandemic in PHC clinics in the City of Johannesburg.

## Research methods and design

### Study design

The study relied on a qualitative, descriptive, phenomenological research design. This approach enabled a comprehensive description of common lived experiences, allowing researchers to free themselves from preconceived assumptions or beliefs.^11^ Phenomenological studies rely on personal reports to understand the human experience described by those concerned.^12^ Phenomenology offers a more in-depth understanding of individuals’ experiences with interest, exploring the essence or meaning of the experience.^12^ This study aimed to develop strategies to enhance and sustain the positive leadership attitudes of operational managers after the COVID-19 pandemic in PHC clinics using a descriptive phenomenological research approach.

### Setting

The study was conducted at PHC clinics in the City of Johannesburg, the largest city in Gauteng province in South Africa. Gauteng has five municipalities, namely the City of Tshwane, the City of Ekurhuleni, Mogale City, Sedibeng District and the City of Johannesburg. The latter consists of seven regions, regions A to G, home to 82 PHC local authority clinics. The research study was conducted at these PHC clinics in the City of Johannesburg.

### Population and sampling strategy

The study population consisted of the 82 operational managers who work at the PHC clinics in the City of Johannesburg. A purposive sampling method was used to select participants who could provide rich, detailed information about the phenomenon to align with the study objectives, and who consented and were willing to participate. The sample ultimately included 45 operational managers who met the criteria of inclusion and voluntarily agreed to participate in the study. The criteria of inclusion were operational managers who worked at PHC clinics in the City of Johannesburg during the COVID-19 pandemic, those willing to participate, those registered with the South African Nursing Council and of any race, age, gender and who could shed light on operational managers’ leadership attitudes after the COVID-19 at PHC clinics in the City of Johannesburg. Participants were recruited during an information session as part of an operational managers’ meeting.

### Data collection method

Data collection was carried out using semi-structured, individual interviews to obtain meaningful and reliable data to explore and describe the leadership attitudes of operational managers after the COVID-19 pandemic at PHC clinics in the City of Johannesburg. A pilot interview was conducted with one of the operational managers to confirm the viability of the interview process. The pilot interview was excluded from the process of data analysis. The research question posed was: ‘How was your leadership attitude post COVID-19 pandemic in the city of Johannesburg metropolitan clinic?’ The question enabled the operational managers to share their viewpoints on their leadership attitudes after the COVID-19 pandemic at PHC clinics in the City of Johannesburg. A date, time and venue were arranged for the interviews according to the participants’ availability. Interviews were conducted at the convenience of participants at times when facilities were not busy and when there were no scheduled meetings for the day. English was the primary language used during the interview. The interviews were audio-recorded with the permission of the participants. The researcher observed the body language of the participants during the interview and documented field notes, creating a clear picture of the operational managers’ attitudes after the COVID-19 pandemic. Privacy, confidentiality and anonymity were ensured with the use of codes (P1, P2, etc.) to ensure participants’ anonymity. Data were secured to avoid unauthorised access, with only the researchers and the independent coder having access.

### Data analysis

The researcher used Colaizzi’s seven-step descriptive phenomenological analysis method to analyse and interpret data to produce meaning, gain an understanding and develop empirical knowledge to summarise the qualitative interviews.^13^

- Step 1: Familiarisation – Audio-recordings were transcribed verbatim. The primary author read and reread the transcripts to gain an understanding of participants’ leadership attitudes after the COVID-19 pandemic.
- Step 2: Identification of significant statement – The author and the independent coder extracted meaningful statements related to the phenomenon, focusing on statements that reveal the leadership attitudes of operational managers after the COVID-19 pandemic.
- Step 3: Meanings formulated – Each meaningful statement from the quotes was analysed to identify underlying meanings and formulate a concise description of the meaning.
- Step 4: Themes clustered – The interpretative meanings were formulated into themes and sub-themes, and patterns and relationships among themes were identified.
- Step 5: Exhaustive description developed – All the clustered themes were analysed, and a detailed description was written of the phenomena, reflecting the fundamental structure of the research topic.
- Step 6: Fundamental structure produced – The primary author captured the aspects deemed essential to the phenomenon to reflect the fundamental structure of the research topic. Meaningful, reliable statements were formulated from the general analysis and information was synthesised to deduce meaning from participants’ experiences with the phenomenon.
- Step 7: Verification sought from the participants – The interview transcripts were forwarded to the participants to enable them to confirm and validate their responses. Meaningful statements were developed regarding participants’ experiences and collected data. The primary author and the independent coder reached consensus on the themes and categories that emerged during the final confirmation meeting, and no amendments were made. The supervisors’ input was sought, and clarity was confirmed.

### Trustworthiness

The researcher adhered to the principles of trustworthiness throughout the entire process.^14^ The veracity of the data was validated through audio-recording, verbatim transcripts, fieldnotes and observations. The researcher thoroughly detailed the research methodology and background to ensure transferability. Regular corrections and feedback meetings with study supervisors helped preserve dependability. Confirmability was guaranteed by back-to-back meetings with the researcher, supervisors and an independent coder to deliberate on findings until consensus was reached.^15^

### Ethical considerations

Permission to conduct the research was obtained from the Research Ethics Committee (REC) at the University of Johannesburg, Faculty of Health Sciences (REC-2072-2023). This procedure ensured that participants’ rights were protected and respected, and that researchers did not act unethically to serve their own interests. Permission to conduct research at PHC clinics in the City of Johannesburg metropolitan municipality was sought from the Johannesburg Health District Research Committee (Jhb DRC) (NHRD REF. NO.: GP_202307_062), which is responsible for coordinating and managing research at clinics in the City of Johannesburg. The researcher adhered to autonomy, privacy, anonymity and confidentiality, beneficence and non-maleficence (risk and benefits) and justice.

## Results

### Demographics of the participants

The study sample consisted of 10 operational managers from among the PHC facilities in different regions of Johannesburg metropolitan. They are presented in Table 1. This consisted of nine female managers and one male manager, with their ages ranging from 35 to 60 years. Of the 10 participants, eight held a diploma in clinical treatment nursing science, health assessment, treatment and care (R48); and the other two held a post-basic BCur qualification in education, administration and clinical treatment nursing science, health assessment, treatment and care.

**TABLE 1:** Summary of participants’ demographic data.

Participant	Age (years)	Gender	Years at the PHC facility
P1	60	Female	12
P2	38	Male	9
P3	42	Female	9
P4	50	Female	10
P5	42	Female	11
P6	45	Female	10
P7	58	Female	18
P8	46	Female	7
P9	35	Female	6
P10	38	Female	8

*Source*: Khakhau et al. Operational managers’ leadership attitudes post COVID-19 pandemic in primary healthcare in the City of Johannesburg metropolitan clinics. University of Johannesburg; 2023.^16^

PHC, primary health care.

Box 1 presents an overview of the themes and sub-themes that emerged from the data analysis. The central theme that emerged was the need for the development of strategies to enhance and sustain the positive leadership attitudes of operational managers after COVID-19.

BOX 1 Outline of the identified theme and sub-themes.

**Table:** 

Theme	Sub-themes
1. Strategies to enhance and sustain the positive leadership attitudes of operational managers after COVID-19	1.1 Debriefing and counselling services 1.2 Teamwork, team-building and senior management support 1.3 Continuous staff development

*Source*: Khakhau et al. Operational managers’ leadership attitudes post COVID-19 pandemic in primary healthcare in the City of Johannesburg metropolitan clinics. University of Johannesburg; 2023.^16^

COVID-19, coronavirus disease 2019.

The COVID-19 pandemic brought unprecedented challenges to PHC, testing the leadership of operational managers like never before. Operational managers who were at the forefront were responsible for guiding their teams through complex health care delivery. However, the pandemic highlighted the need for positive leadership attitudes to prioritise resilience, adaptability and empathy. As the PHC sector must be rebuilt and recover, it has been essential to develop and sustain leadership attitudes that support the well-being of both patients and staff. Positive leadership attitudes have the potential to influence those around the manager to remain calm, bringing out feelings of optimism, collaboration and a sense of ease. The strategies to enhance and sustain positive leadership attitudes are discussed below.

### Sub-theme 1.1: Debriefing and counselling services

Participants deemed the need for debriefing and counselling services urgent and vital. It plays a critical role in supporting the emotional and psychological well-being of health care staff members. Debriefing and counselling can provide a safe space for health care workers to process their emotions and fears after the COVID-19 pandemic, helping them to deal with pandemics better in future.^6^ Sharing their experiences allows a sense of unity and understanding. Debriefing will help identify lessons learned, best practices and areas of improvement. Participants considered debriefing important for health care professionals to remain positive.

‘Have debriefing sessions within the facility, they need support from senior management. They must come to our clinics a just for support visit us, to show they care about the staff, and it will encourage them, or it will boost them their morale and encourage the staff to go for employee wellness programs.’ (P8, female, 46-years-old)

‘The pandemic has put us through a lot, we were also afraid that we were going to die, and we lost some of our colleagues and we were not even able to go to their funerals due to all the restrictions that came with the pandemic. We are still challenged mentally we need debriefing; EAP need to step in now.’ (P2, male, 38-years-old)

It is evident from the above quotations that the pandemic left an indelible mark on health care professionals, particularly the operational managers who bore the brunt of the crisis. Prolonged exposure to high stress and a traumatic work environment took a significant toll on the mental health and well-being of everyone, especially the operational managers. To address this, participants expressed the need for an employee assistance programme (EAP) for early identification of distressed employees through an evaluation of declining job performance from others. Debriefing is aimed at understanding past experiences, helping those who have been traumatised to regain functioning after the incident. The participating operational managers from the PHC clinics in the City of Johannesburg PHC expressed that they were exhausted and needed some form of counselling to enable them to provide debriefing to their subordinates.

They said the following:

‘We really need the debriefing services because I’m sure there are still those who have not dealt with wherever we came from. The need for debriefing services will enable people to view their fears to say whatever they were not able to say in that time of COVID. And I think if those services will allow every, every employee, including us as management and it will it make us have psychologically readiness for work, psychologically improved and psychologically awakening.’ (P3, female, 42-years-old)

‘We can also debrief one another as staff members, through team-building games. But we need to make time away from the facility. We go through a lot whether personal or professional.’ [*Taking a deep breath*] (P4, female, 50-years-old)

Participants confirmed that the COVID-19 pandemic exposed many of them to an unfamiliar environment, leaving them with a crisis of adaptability and the need for clinical debriefing and counselling to cope. Debriefing can ensure that the victim reviews the incident, reflects and learns from it.^17^ This helps the victims to find solutions for their problems and to get support after the incident. Debriefing can provide a safe space for operational managers and their subordinates to share their stories, reflect on their experiences and gain perspective on their challenges. Counselling services can offer support to address anxiety, depression, post-traumatic stress disorder and other mental health concerns.

### Sub-theme 1.2: Teamwork, team-building and senior management support

Participants noted the importance of teamwork, team-building and senior management support in health care. Effective teamwork is instrumental in ensuring quality service delivery, managing workload pressure and managing staff morale. Teamwork can also improve work efficiency, allowing group members to combine problem-solving ideas in an organisation. Teamwork is one of the essential elements that leads to the achievement of organisational goals and vision; it is important for the prosperity of an organisation.

‘Teamwork and teambuilding for our facility is important, one nurse cannot go home overwhelmed, while others are relaxed, team efforts are important for quality patient care, and everyone has an opportunity to learn from others.’ (P5, female, 42-years-old)

The above shows that the participants valued the importance of teamwork and team-building, which help to foster a sense of unity and shared purpose. This enabled them to work together seamlessly and support one another through challenging times. They further stated that effective team-building promotes open communication, creative problem-solving and allows teams to respond effectively to challenges and changes. Organisations need to invest in team-building activities and initiatives to improve staff morale, reduce turnover and enhance staff overall performance. Operational management can implement cost-effective strategies that can be beneficial to their respective teams by scheduling regular team meetings and debriefing sessions to discuss challenges, successes and areas of improvement. Others suggested the creation of social events and activities to promote team bonding and mutual trust, such as team lunches, volunteer days and team outings.

‘Madam, anything that is going to motivate you talking about monetary and non-monetary, you see having lunch one day, all of us at work organising for a lunch. Now it’s December time. Team building, this is what you call a team being going out for the can be team building. You know, you are going to do team building. Or they are giving us something like one during the nurses’ day. They used to give us presents. They used to give us this. Now it’s totally real. There is nothing. So, everything revolves around motivation.’ [*Opening her diary*] (P5, female, 42-years-old)

The participants further expressed the need for support from senior management in providing operational managers with personnel, guidance and the autonomy they need to lead the team effectively. They noted that a more visible executive presence could help promote a culture of transparency, trust and open communication, empowering operational managers to make decisions, innovate and remain resilient. By prioritising teamwork, team-building and senior management support, health care organisations can build resilient, high-performing teams that are better equipped to navigate the complexities and uncertainties of modern health care.

Participants said the following:

‘It feels like you are alone as a manager without support from senior management instead they would call only if there [?] Is a problem or when they want stats. We needed support to deal with issues of shortage of staff, PPE, staff absenteeism and shortage of swabs and I had to be answerable to the employees.’ [*Looking at the door*] (P8, female, 46-years-old)

‘Senior management is important to help us manage these facilities better; management should not only come when there is a crisis but also come to give us support and just to encourage and reassure the staff. You know our working conditions are not that conducive.’ (P1, female, 60-years-old)

Senior management support provides guidance and vision for the facility, ensuring alignment with the organisational goals. Senior management support comes in many forms, including effective communication, promoting workers’ involvement in crucial decision-making and giving employees clear feedback on their performance to ensure quality work. Support results in innovative work behaviour, creates recognition of employees’ arduous work and ensures that the employer recognises employees’ well-being. By providing effective support, senior management can play a critical role in promoting the success and sustainability of primary health care clinics.

### Sub-theme 1.3: Continuous staff development

The participants expressed the need for continuous development as one of the strategies to enhance and sustain a positive leadership attitude of operational managers. However, the need for continuous development is not only beneficial for the health care workers but also for quality patient care. By prioritising staff development, primary health care clinics can promote the culture of excellence, innovation and continuous improvement, ultimately enhancing patient care and outcomes.

‘So, I think the strategies that came up during COVID-19 pandemic are the need for more training, staff development boosts the confidence and morale of the staff. Even so imagine being a manager who is clueless.’ [*Picking up a glass of water*] (P4, female, 50-years-old)

‘Encourage employees to do online courses from knowledge hubs. It also gives you the CPD points if we can, we do not have to wait for training provided by the city. Do online courses to expand your knowledge and do not all go for post basic courses the list is long.’ (P6, female, 45-years-old)

Operational managers and subordinates need to have a plan for improving the existing knowledge and skills of subordinates and to develop a skills development and training programme according to employees’ needs.^1^ Operational managers also emphasised the importance of developing their leadership skills and the need for leadership courses. Participants’ reflections showed that continuous staff development is crucial in enhancing skills and knowledge, ensuring they provide high-quality care. Staff development is also linked to improved patient outcomes, as health care professionals are better equipped to manage complex cases and provide evidence-based care. Staff development helps health care professionals adapt to the ever-changing health care landscape and technological advancements, fostering innovation and improvement.

Other participants mentioned:

‘Staff development is needed to advance leadership skills that will assist us as managers. You know we also need to learn how to cope with situations. And learn how to cope as a manager in times of a crisis or pandemic.’ (P1, female, 60-years-old)

‘Development of staff is Important to ensure that clinicians and other health personnels are well vested in terms of new guidelines and protocols.’ (P8, female, 46-years-old)

Participants expressed the need to overcome previous challenges regarding staff development, such as ensuring that staff development is aligned with organisational goals and priorities, and the allocation of staffing and budget to support initiatives. There are also time constraints, necessitating flexible staff development opportunities such as online learning and time off work to attend courses. Operational managers should also communicate the importance of staff development, addressing concerns and resistance to change.

## Discussion

The findings of this study revealed that the leadership attitudes of operational managers remained positive even after the COVID-19 pandemic, as they continued to manage the facilities with positive attitudes. Some of the things they learned during the pandemic had a positive effect on their leadership after the COVID-19 pandemic. It became evident that despite the challenges they faced, they learned to adapt and became flexible and resilient during the pandemic. They learned that leaders need a combination of leadership traits to achieve organisational goals and help mitigate adverse responses to crisis management.^18^ However, while they did learn lessons from the pandemic about leadership,^19^ the operational managers discovered the importance of being attentive and listening to their subordinates, including the creation of a transparent relationship with their subordinates.

Findings further revealed that participants experienced challenges to positive leadership that can hinder the development of positive leadership attitudes. By addressing these and implementing supportive strategies, primary health care clinics can cultivate positive leadership, improve patient care and enhance the overall health care experience.^20^ Operational managers declared that the shortage of equipment led to negative staff attitudes, low morale and a lack of staff incentives and appreciation. The shortage can have a profound impact on operational managers, compromising their ability to effectively lead and manage their teams^21^ with impairment of cognitive function, judgement and decision-making abilities, ultimately affecting the quality of care and service provided.^22^

Furthermore, the operational managers acknowledged increased stress, anxiety and depression, which can spill over into their personal lives, affecting relationships and overall well-being.^23^ Staff shortage can severely hinder management, leading to compromised service delivery, decreased productivity and increased risks. Insufficient staffing can result in overwhelming workloads, burnout and decreased staff morale, ultimately affecting the quality of care and service provided.^24^

The participants recommended the formulation of strategies to enhance and sustain the positive leadership attitudes of operational managers, adding that these strategies will empower and inspire them. Enhancing and sustaining positive leadership attitudes among operational managers is critical for quality patient care, motivating teams and navigating complex challenges.^25^ Investing in leadership development programmes will result in better well-being and self-care, empowered operational managers, effective communication and a positive leadership culture.^26^ Staff development is important in ensuring quality nursing care, keeping abreast with the current nursing trends and ensuring that employees have mentors to help them grow in the profession.^27^ Leadership courses will help operational managers to improve leadership skills such as strategic thinking, communication and problem-solving.^28,29,30^

The COVID-19 pandemic has also highlighted the importance of prioritising the mental health and well-being of managers. Services, such as debriefing and counselling, were affirmed as essential components of a comprehensive approach to supporting the mental health and well-being of individuals.^31^ The provision of accessible services to health care professionals can help manage the negative effects of their work, rebuild their resilience and maintain their mental health and well-being.^32^ Debriefing and counselling will allow individuals affected by the pandemic to deal with the traumas and provide a safe space to process their emotions and reactions following stressful events.^33,34,35,36^

The managers mentioned teamwork, team-building and senior management support as vital to enhance and sustain positive leadership attitudes. These strategies have been identified as essential components of high-performing organisations. They would help facilitate clear goals and expectations, provide opportunities for team-building and socialisation, and recognise and reward team achievements to boost staff morale and better outcomes for individuals.^37^ Teamwork and team-building^38^ are essential components of achieving success in an organisation, particularly in health care. Ultimately, they are critical investments for any organisation seeking to achieve excellence.^39^ When team-building is part of the culture in health care, it facilitates team efforts, helps co-workers know each other’s strengths and weaknesses, and helps to engage more and understand one another.^40^ Managers thus expressed the need for time and sufficient staffing to facilitate team-building activities.^28,41,42,43,44,45,46^

## Limitations of the study

The study was limited to the leadership attitudes of operational managers and excluded other multidisciplinary team members in PHC, such as medical doctors, clinicians and senior management. Some participants were not comfortable sharing what hinders them from retaining their positive leadership attitudes. The availability of limited literature dealing with the leadership attitudes of operational managers at PHCs in the City of Johannesburg was evident, and participants had limited time for interviews because of their busy schedules. Some of the participants were unavailable for interviews because of their demanding work schedule and could not reschedule.

## Recommendations

The Gauteng Department of Health (GDoH) should establish an effective Employee Wellness assistance programme (EAP) that is easily accessible and responsive in times of pandemics such as COVID-19 and other unforeseen disasters. Operational managers and senior managers should endeavour to create a conducive work environment that encourages a culture of trust and openness. Interpersonal relationships among co-workers should be sustained through teamwork and team-building opportunities. Conflict in the workplace should be efficiently managed, with mediation between co-workers and disputes managed precisely to improve teamwork and create a happier workforce. Employees should create a self-directed development plan for their skills development. Senior management should invest in team-building opportunities for any organisation seeking to achieve excellence, innovation and success. The Central Education and Training Units (CETU) ought to develop continuous training programmes and should encourage the employees to use platforms such as the knowledge hub for online training.

## Conclusion

This study provided insight into the need for strategies to enhance and sustain the positive leadership attitudes of operational managers. Participants expressed the need for these strategies as they faced challenges and changes in the health care system. They affirmed that these challenges would enable them to influence employee morale and productivity. The interviews revealed a lack of senior management support, resulting in operational managers feeling overwhelmed and isolated during times of need. There were recommendations for attention. The study contributed to the understanding of the much-needed strategies for the leadership attitudes of operational managers in the PHC clinics in the City of Johannesburg.
